# Host long noncoding RNAs in bacterial infections

**DOI:** 10.3389/fimmu.2024.1419782

**Published:** 2024-09-02

**Authors:** Yong Cheng, Yurong Liang, Xuejuan Tan, Lin Liu

**Affiliations:** ^1^ Department of Biochemistry and Molecular Biology, Oklahoma State University, Stillwater, OK, United States; ^2^ Oklahoma Center for Respiratory and Infectious Diseases, Oklahoma State University, Stillwater, OK, United States; ^3^ Department of Physiological Sciences, Oklahoma State University, Stillwater, OK, United States

**Keywords:** long noncoding RNAs, host-pathogen interactions, bacterial infections, immunity, host cells

## Abstract

Bacterial infections remain a significant global health concern, necessitating a comprehensive understanding of the intricate host−pathogen interactions that play a critical role in the outcome of infectious diseases. Recent investigations have revealed that noncoding RNAs (ncRNAs) are key regulators of these complex interactions. Among them, long noncoding RNAs (lncRNAs) have gained significant attention because of their diverse regulatory roles in gene expression, cellular processes and the production of cytokines and chemokines in response to bacterial infections. The host utilizes lncRNAs as a defense mechanism to limit microbial pathogen invasion and replication. On the other hand, some host lncRNAs contribute to the establishment and maintenance of bacterial pathogen reservoirs within the host by promoting bacterial pathogen survival, replication, and dissemination. However, our understanding of host lncRNAs in the context of bacterial infections remains limited. This review focuses on the impact of host lncRNAs in shaping host−pathogen interactions, shedding light on their multifaceted functions in both host defense and bacterial survival, and paving the way for future research aimed at harnessing their regulatory potential for clinical applications.

## Introduction

One of the major breakthroughs in molecular biology over the last few decades has been the discovery and demonstration of long noncoding RNAs (lncRNAs). LncRNAs are a large and relatively novel class of RNAs transcribed from the genome, with a length of >200 nucleotides, and are generally characterized by the lack of protein-coding potential (1). LncRNAs function through a variety of mechanisms via their interactions with DNA, RNA and proteins, regulating various cellular processes in humans and animals (2, 3). Research investigating the engagement of host lncRNAs in host−pathogen interactions during bacterial infections is still emerging. In this review, we briefly discuss the role of host lncRNAs in immunity and then focus on the functional role and mechanisms of host lncRNAs in response to bacterial infections. Finally, we discuss the potential application of host lncRNAs and a perspective on lncRNA research in the context of bacterial infections.

## Discovery of host lncRNAs

The vast amounts of DNA between protein-coding genes were considered “junk” DNA in the 1970s (4). Two decades later, the discovery of lncRNAs and the identification of their biological functions revolutionized our view of this “junk’ DNA. In the 1980s, differential hybridization screening of eukaryotic cDNA libraries was used to study tissue-specific genes with a temporal pattern of expression. The first lncRNA, *H19*, was identified by screening a murine fetal liver cDNA library (5, 6). This lncRNA exhibits high sequence conservation between humans and mice and plays an important role in embryonic development (7, 8). The *H19* locus and adjacent gene insulin-like growth Factor 2 (*Igf2*) are reciprocally imprinted and located on chromosome 7 in mice and chromosome 11p15.5 in humans. The expression of *H19* and *Igf2* is regulated by an intergenic differentially methylated region (DMR) upstream of *H19* and several tissue-specific enhancers downstream of the *H19* gene. As a result, *H19* is exclusively expressed from the maternal chromosome, whereas *Igf2* is expressed from the paternal allele (9). *H19* was initially thought to be an mRNA until the discovery of another lncRNA, X-inactive-specific transcript (*Xist*), in the early 1990s. The *Xist* is critical for X chromosome inactivation in female mammals (8, 10, 11). Unlike *H19*, *Xist* has poor primary sequence conservation between humans and mice. *Xist* triggers gene silencing in *cis* in the X chromosome through the chromatin-binding region in female cells. This in turn catalyzes a cascade of chromatin changes and global spatial reorganization, ultimately leading to the silencing of the entire chromosome. Since then, *H19* and *Xist* have been widely investigated, and dysregulation of these two lncRNAs has been shown to be involved in various human diseases and conditions, such as tumorigenesis, nervous system diseases, aging and inflammation (12–14).

LncRNAs have attracted increasing attention as a result of the completion of the Human Genome Project (HGP). One of the greatest advantages of HGP is the discovery that the number of protein-coding genes is far less than expected and that less than 2% of the human genome represents protein-coding regions (15–17). This was confirmed by the Encyclopedia of DNA Elements (ENCODE) project, which includes even larger-scale studies conducted in humans and mice (18, 19). Through various techniques, such as microarray hybridization and deep sequencing analyses, it is estimated that 70–90% of the human genome is transcribed, including protein-coding and noncoding RNAs, at some points during development (20–23). The rapid development of high-throughput DNA sequencing technologies and deep RNA sequencing for eukaryotic transcriptomes has led to an explosion in the number of newly identified and uncharacterized lncRNAs (24–26). Although there are arguments regarding poor conservation between humans and mice, shorter lifespans and fewer copies of lncRNAs than of mRNAs (27), it is becoming increasingly clear that lncRNAs have ubiquitous biological functions in translational inhibition, mRNA degradation, RNA decoys, the recruitment of chromatin modifiers, the regulation of protein activity and the availability of microRNAs via a sponging mechanism (28). However, considering lncRNA:microRNA stoichiometry, the extent to which lncRNAs function as sponges for microRNAs is a concern, particularly for those lncRNAs with low abundance. LncRNAs have been detected in various human body fluids and are potential diagnostic biomarkers for human diseases (29, 30). Additionally, an increasing number of studies indicate that lncRNAs are potential drug targets for the development of novel therapies for human diseases (3, 31).

## Role of host lncRNAs in immunity

**Figure 1:**
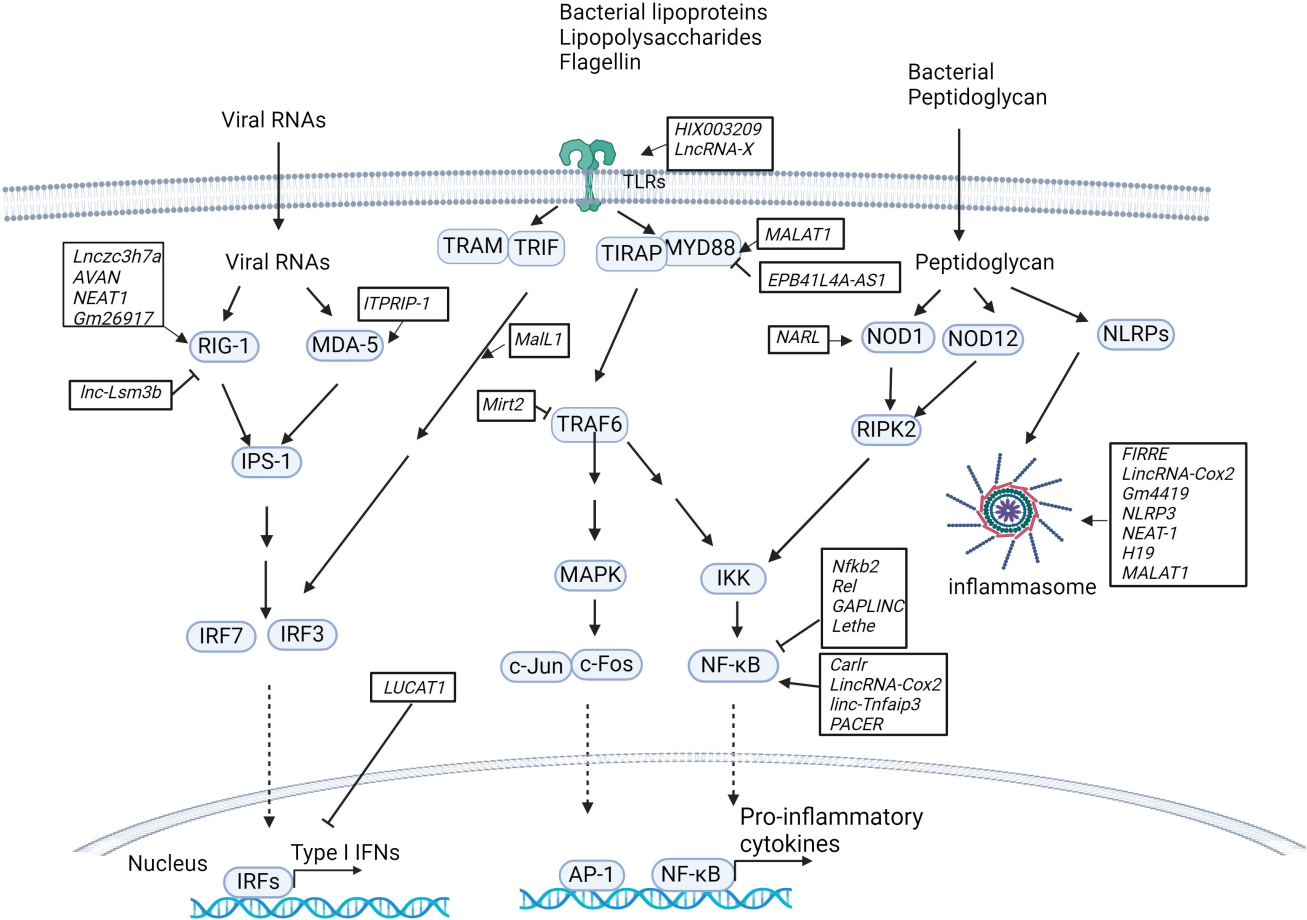
LncRNAs in innate immunity pathways. Host lncRNAs are engaged in various signaling pathways and cellular responses to invading microbial pathogens in immune cells. LncRNAs regulate the RIG-I/MDA-5-dependent nucleic acid-sensing pathways that recognize microbial RNAs in the cytosol of host cells and subsequently activate the expression of type I IFNs. LncRNAs are also important for TLR-dependent pathways in response to microbial infections and activate the expression of proinflammatory cytokines and type I IFNs. Additionally, host lncRNAs also regulate other cellular responses, such as NOD1/NOD12-dependent pathways and NLRP-dependent inflammasome activation.

Accumulating evidence suggests that lncRNAs play key roles in the proliferation, differentiation and activation of immune cells, including monocytes, macrophages, dendritic cells (DCs), neutrophils, granulocytes, T cells and B cells. Dysregulated expression of lncRNAs leads to aberrant immune responses in infectious diseases (**Figure 1**) (32, 33). The engagement of lncRNAs in immunity has been well described in previous reviews (34–36). In the following section, we provide a concise overview of the role of lncRNAs in the host immune response.

### LncRNAs in TLR-dependent pathways

Recent studies have revealed that lncRNAs constitute an essential part of the network involved in toll-like receptor (TLR) signaling pathways by acting as competitive endogenous RNAs (ceRNAs) that sponge microRNAs, directly interacting with transcription factors and subsequently activating or inhibiting the expression of downstream genes, regulating protein ubiquitination and activity, or mediating epigenetic chromatin remodeling (**Figure 1**). For example, by sponging miR-6089, the lncRNA *HIX003209* restores the expression of TLR4 and the activation of the downstream signaling molecule NF-κB in human macrophages (37). The *lncRNA XIST* sponges miR-154-5p, which inhibits the expression of TLR5 in a rat neuropathic pain model (38). The lncRNA *MALAT1* upregulates MyD88 by sponging miR-149 to promote LPS-induced inflammatory responses in a lung injury model (39). Lack of lncRNA *EPB41L4A-AS1* expression reduces the enrichment of H3K9me3 in the MYD88 promoter region and subsequently promotes activation of the NF-κB pathway in human peripheral blood mononuclear cells (PBMCs) (40). The LPS-inducible lncRNA *Mirt2*, which serves as a repressor of inflammatory responses, negatively regulates TLR4 signaling. *Mirt2* restricts the MyD88-dependent MAPK and NF-κB signaling cascades by interacting with TRAF6 and subsequently inhibiting TRAF6 oligomerization and ubiquitination in mouse macrophages (41).

In addition to the lncRNAs described above, several mammalian lncRNAs have been found to regulate the transcription factor NF-κB, a critical component of TLR-dependent pathways (**Figure 1**). These lncRNAs can serve as repressors or activators under various conditions. LncRNAs act as repressors: i) LncRNA *Nfkb2* and lncRNA *Rel* regulate the innate immune response upon LPS stimulation in mouse bone marrow-derived macrophages (BMDMs) by binding to the transcription factors NF-κB, IRF3, JunB and c-Jun (42). ii) lncRNA *Lethe* exerts an anti-inflammatory effect by binding to the p65 subunit of NF-κB and blocking its binding to target promoters and the subsequent expression of downstream genes (43). iii) Gastric adenocarcinoma predictive long intergenic noncoding RNA (lncRNA *GAPLINC*), a lncRNA that was initially found in cancer progression, is highly expressed during human and mouse macrophage differentiation. A recent study indicated that the lncRNA *GAPLINC* downregulates innate immunity in response to endotoxic shock induced by bacterial lipopolysaccharide (LPS) (44). LncRNAs act as activators: i) lncRNA *Carlr* promotes the expression of NF-κB-regulated genes via interactions with the p65 subunit of NF-κB in human THP-1 cells (45). ii) *LincRNA-Cox2* (also called Ptgs2os2) is known to regulate immune gene expression in immune cells. RNA-seq analysis revealed that *lincRNA-Cox2* is one of the most highly induced lncRNAs following TLR2, TLR4 and TLR7/8 stimulation in mouse BMDMs. *LincRNA-Cox2* regulates the expression of inflammatory genes by binding to heterogeneous nuclear ribonucleoproteins hnRNP-A/B and A2/B1 (46). It has also been reported that *lincRNA-Cox2* is assembled into the switch/sucrose nonfermentable (SWI/SNF) complex after LPS stimulation in mouse macrophages, where it mediates SWI/SNF-associated chromatin remodeling and NF-κB activation (47). iii) Similarly, p50-associated COX-2 extragenic RNA (PACER) regulates COX-2 expression by interacting with the repressive subunit of NF-κB, p50, and thus activating the NF-κB pathway in human mammary epithelial cells and monocytes/macrophages (48). iv) *LincRNA-Tnfaip3* interacts with high-mobility group Box 1 (HMGB1), which facilitates nucleosome remodeling and the accessibility of genomic DNA to the transcription factor NF-κB subunit p50 in mouse macrophages (49).

Additional lncRNAs that are involved in TLR-dependent signaling and the regulation of downstream gene expression have been identified. i) Myristoylated alanine-rich C kinase substrate (MARCKS) cis regulating the lncRNA promoter of cytokines and inflammation (*MROCKI*), also known as the regulator of cytokines and inflammation (*ROCKI*), acts as a master regulator of inflammatory responses through reducing MARCKS transcription and inflammatory gene expression in multiple TLR-stimulated human macrophages (48, 50). ii) Macrophage interferon-regulatory lncRNA 1 (*MalL1*) acts as an architectural RNA component of the TLR4-dependent pathway and stabilizes optineurin, a ubiquitin-adapter platforming TBK1 kinase, thus regulating TLR4–TRIF–IRF3-mediated type I interferon (IFN) expression in human macrophages (51). iii) LncRNA *LUCAT1* is an inducible lncRNA that is localized primarily within the nucleus, where it functions as a negative feedback inhibitor of type I IFN and inflammatory gene expression by regulating the splicing and stability of nuclear receptor 4A2 (NR4A2) in human myeloid cells (52, 53). iv) LncRNAIL7–AS is a TLR2/TLR4-responsive gene that positively regulates the expression of inflammatory genes by modulating histone acetylation via interaction with the transcriptional coactivator p300 and assembly of SWI/SNF chromatin remodeling complexes at the promoter regions of these genes (54). v) The miR-155 host gene (*MiR155HG*) encodes a precursor RNA of microRNA-155 (miR-155) and lncRNA-155 in mice and humans. Previous studies have focused mainly on the role of miR-155 in inflammation and TLR-dependent pathways (55). More recently, the second product of *MiR155HG*, lncRNA-155, was shown to positively regulate the host response in immune cells and mice in the context of IAV infection (56). In contrast to that of miR-155, the role of lncRNA-155 in immunity remains to be defined. Additionally, determining the crosstalk between miR-155 and lncRNA-155 and understanding their unique roles in the host response to infections would be interesting.

### LncRNAs in NLR-dependent pathways

NOD-like receptors (NLRs, also known as nucleotide-binding leucine-rich repeat receptors) play a key role in inflammatory and apoptotic responses by recognizing a variety of pathogen-associated molecular patterns (PAMPs) from microbial pathogens, such as bacterial peptidoglycan, flagellin and viral RNA. Host lncRNAs regulate NLR abundance and the formation and activation of inflammasomes (**Figure 1**). For example, by sponging miR-217-5p, the lncRNA *NARL* regulates the abundance of the NOD1 protein and, thus, downstream signaling in teleost fish (57). The lncRNAs *FIRRE*, *lincRNA-Cox2* and *Gm4419* bind and activate NF-κB and form a positive feedback loop to promote the activation of the NLRP3 inflammasome in cerebral microglia (58). Suppression of the lncRNA *NLRP3* inhibits NLRP3-triggered inflammatory responses in early acute lung injury (59). The lncRNA nuclear paraspeckle assembly transcript 1 (lncRNA NEAT1) enhances NLRP3, NLRC4, and AIM2 inflammasome assembly and promotes caspase-1 activation, cytokine production, and pyroptosis in mouse macrophages (60). The lncRNA *H19* significantly promotes NLRP3/6 inflammasome imbalance by sponging miR-21 to facilitate programmed cell death 4 (PDCD4) expression in mouse microglia (61). The lncRNA *MALAT1* inhibits Nrf2 by regulating EZH2-mediated epigenetic repression, thereby increasing ROS levels and inflammasome activation in a Parkinson’s disease mouse model (62). The myocardial infarction-associated transcript (lncRNA *MIAT*) suppresses inflammasome-induced macrophage pyroptosis by inhibiting the expression of IL-1β and TNFα (63).

### LncRNAs in RLR-dependent pathways

RIG-I-like receptors (RLRs) are key nucleic acid sensors that detect virus infections in immune cells (64). It has been shown that lncRNAs target the RLR-mediated signaling pathway, regulating the antiviral host response within immune cells, although these studies were performed in the context of virus infection. Some lncRNAs positively modulate the RLR signaling pathway by mediating the expression of type I IFNs (**Figure 1**). For example, the lncRNA *Lnczc3h7a* binds to both TRIM25 and RIG-I, serving as a molecular scaffold for stabilizing the RIG-I-TRIM25 complex and promoting RIG-I-mediated antiviral responses in mouse peritoneal macrophages (65). The lncRNA *AVAN* also binds to TRIM25 and promotes the interaction between TRIM25 and RIG-I. Additionally, the lncRNA *AVAN* positively regulates FOXO3a expression by remodeling the FOXO3a promoter region, facilitating neutrophil chemotaxis and recruitment in influenza A virus (IAV)-infected patients (66). The lncRNA *NEAT1* is induced by virus infection, such as influenza virus, HSV, Hantaan virus (HTNV) and SARS-CoV-2 virus (67–69). *NEAT1* interacts with the splicing factor SFPQ and upregulates the expression of RIG-I (70). *Gm26917* reduces the degradation of RIG-I mRNA by sponging miR-124-3p in SARS-CoV-infected cells (67). The lncRNA *ITPRIP-1* binds to the C-terminus of MDA5, another cytosolic RNA sensor, and promotes MDA5 oligomerization and activation in hepatitis C virus-infected cells (71). Some lncRNAs serve as negative regulators of the RLR-mediated signaling pathway, leading to the inhibition of the antiviral response. For example, *lnc-Lsm3b* inhibits RIG-I activation by competing with viral RNAs for the binding of RIG-I monomers and therefore attenuates the RIG-I-mediated antiviral response in vesicular stomatitis virus-infected mouse macrophages (72).

## LncRNAs in host−pathogen interactions during bacterial infections

As described above, lncRNAs regulate cellular pathways that are involved in various biological processes, including host−pathogen interactions during bacterial infections caused by *Mycobacterium tuberculosis*, *Salmonella typhimurium*, *Listeria monocytogenes* and *Pseudomonas aeruginosa*. In the following sections, we discuss the role of host lncRNAs in host–bacteria interactions and their potential applications as novel diagnostic biomarkers and in host-directed therapy (**Table 1**).

**Table 1:** Host lncRNAs in bacterial infections.

LncRNA Name	Host Cells	Bacterial Pathogens	Effects	Mechanism of Action	References
*NEAT1*	THP-1-derived macrophages	*M. tuberculosis*	Inhibit *M. tuberculosis* survival in macrophages	Unclear	(73)
*HOTAIR*	THP-1-derived macrophages	*M. tuberculosis*	Inhibit *M. tuberculosis* survival in macrophages	Repress the production of DUSP4 and SATB1	(74)
*lincRNA-Cox2*	RAW 264.7 cells	*M. tuberculosis*	Inhibit *M. tuberculosis* survival in macrophages	Unclear	(75)
*Lnc-EST12*	RAW 264.7 cells and mouse BMDMs	*M. tuberculosis*	Downregulate the expression of proinflammatory cytokines and activation of the NLRP3 inflammasome and GSDMD pyroptosis-IL-18 immune pathway in macrophages during *M. tuberculosis* infection	Interact with transcription factor FUBP3	(76)
*PCEED1B-AS1*	THP-1-derived macrophages and human MDMs	*M. tuberculosis*	Regulate apoptosis and autophagy in macrophages	Act as a sponge for miR-155	(77)
*lincRNA-Cox2*	RAW 264.7 cells	*M. bovis BCG*	Inhibit *M. bovis* BCG-induced apoptosis in macrophages	Unclear	(78)
*IncRNA-MIAT*	THP-1-derived macrophages	*M. bovis BCG*	Repress autophagy and apoptosis in macrophages	Negatively regulate the miR- 665/ULK1 axis	(79)
*IncRNA-MEG3*	THP-1-derived macrophages	*M. bovie BCG*	Facilitate *M. bovis* BCG survival in macrophages	Inhibit autophagy	(80)
*IncRNA-EPS*	RAW 264.7 cells	*M. bovie BCG*	Regulate apoptosis and autophagy in macrophages	Via the KNK/MAPK-dependent pathway	(81)
*NeST*	CD8^+^ T cells	*S. typhimunium*	Increase host resistance to *S. typhimunium* infection	Enhance IFN-γ expression in CD8^+^ T cells by interacting with WDR5	(82)
LncRNA *NEAT1* variant 2	Hels cells	*S. typhimunium*	Increase host resistance to *S. typhimurium* infection	Regulate the the expression of immune-related genes (*TNFSF9*, *CCL2* and *CSF1*)	(83)
*AS-IL1α*	Mouse BMDMs	*L. monocytogenes*	Drive *IL- 1a* expression	Facilitate the RNA Polymerase II (RNAPII) to the *IL- 1a* promoter	(46, 84)
*Sros1*	Mouse BMDMs	*L. monocytogenes*	Inhibit the expression of immune-related genes	Bind to and destabilize the mRNA of the transcription factor *Stat1*	(85)
*lincRNA-EPS*	Mouse BMDMs	*L monocytogenes*	Contribute to *L. monocytogenes* survival within the host	Interfere with the expression of proinflammatory cytokine genes	(86)
*MEG3-4*	MH-S cells and human epithelial cells	*P. aeruginosa*	Contribute to *P. aeruginosa* survival within the host	Regulate the production of the IL-1β protein via acting as a sponge of miRNA-138	(87, 88)
*MalL1*	Human MDMs	*L. pneumophila*	Contribute to host defense against *L. pneumophila*	Upregulate the expression of type I IFNs	(51)
LncRNA *Gm 28309*(Mice) and IncRNA *P33714* (Humans)	RAW 264.7 cells and THP-1-derived macrophages	*B. abortus*	Contribute to *B. abortus* survival within macrophages	Downregulate the activation of NLRP3 inflammasome	(89)

### Mycobacterium tuberculosis

*Mycobacterium tuberculosis* (*M. tuberculosis*) is an intracellular bacterial pathogen that causes tuberculosis (TB), one of the deadliest infectious diseases in humans. According to a World Health Organization (WHO) report, *M. tuberculosis* has infected approximately one-third of the world’s population, leading to ~1.5 million active TB cases and ~1.0 million deaths annually (90). TB is transmitted primarily through the inhalation of respiratory droplets containing *M. tuberculosis*. The transmission of TB occurs when an infected individual expels these droplets into the air through activities such as coughing, sneezing, talking, or even singing. Upon inhalation, *M. tuberculosis* is engulfed primarily by alveolar macrophages, resulting in three distinct consequences: clearance and active or latent infection (91–93). The engagement of host lncRNAs in each infection stage remains to be defined. However, increasing evidence indicates that host lncRNAs play important roles in host*–M. tuberculosis* interactions in macrophages, the primary host cells involved in *M. tuberculosis* intracellular survival. The expression of several host lncRNAs is differentially regulated in human (94, 95) or mouse (96, 97) macrophages following *M. tuberculosis* infection in cell culture. Differential expression of host lncRNAs was also detected in peripheral blood samples from patients with active pulmonary TB compared with healthy controls (98, 99). The role of the majority of these differentially expressed host lncRNAs in host–*Mycobacteria* interactions remains unclear.

### The lncRNA NEAT1

The lncRNA *NEAT1* regulates multiple cellular processes, including gene regulation, the stress response, and immune modulation, by interacting with various proteins and miRNAs in mammalian cells. As described above, *NEAT1* is involved in the formation of nuclear bodies called paraspeckles by serving as a scaffold for several proteins, including splicing factor, proline, glutamine rich (SFPQ), non-POU domain-containing cctamer-binding protein (NONO), polypyrimidine tract-binding protein-associated splicing factor (PSF), fused in sarcoma (FUS), RNA-binding fox-1 homolog 2 (RBFOX2) and matrin 3 (MATR3) (100). In addition to interacting with proteins, NEAT1 can also interact with miRNAs, such as miR-377, by acting as a “sponge” or “decoy” (101). The expression of NEAT1 has been reported to be upregulated in human THP-1-derived macrophages after *M. tuberculosis* infection in cell culture, and this host lncRNA is crucial for controlling *M. tuberculosis* replication within macrophages (73). The mechanism of action of *NEAT1* in macrophages during *M. tuberculosis* infection remains unclear.

### The lncRNA HOTAIR

The lncRNA HOX transcript antisense RNA (*HOTAIR*) is known for its involvement in epigenetic regulation and its impact on gene expression patterns (102, 103). The *HOTAIR* gene is located within the *HOXC* gene cluster on chromosome 12 and is transcribed from the opposite strand of the *HOXC* locus. In a recent study, Subuddhi et al. reported that the expression of the lncRNA *HOTAIR* is downregulated in virulent *M. tuberculosis* H37Rv-infected human THP-1-derived macrophages compared with that in cells infected with avirulent *M. tuberculosis* H37Ra. Furthermore, the lncRNA *HOTAIR* inhibits the production of dual-specific MAP kinase phosphatase 4 (DUSP4) and specific AT-rich sequence binding protein 1 (SATB1), two host proteins that facilitate the survival of virulent *M. tuberculosis* in THP-1-derived macrophages, by increasing the trimethylation of histone H3 at lysine 27 (H3K27me3) around the transcription start sites (TSSs) of these two genes (74). Therefore, this study suggests that *M. tuberculosis* has evolved a mechanism for its intracellular survival by downregulating the lncRNA *HOTAIR*-mediated antimycobacterial activity in macrophages.

### LincRNA-Cox2

*LincRNA-Cox2* is located on chromosome 1 and is transcribed from the antisense strand in mice. The mature sequence of *lincRNA-Cox2* comprises 2 exons and spans a length of 1.7 kb. Its closest neighboring protein-coding gene, prostaglandin-endoperoxide synthase 2 (*Ptgs2* or *Cox2*), is located 50 kb upstream and is transcribed from the sense strand (104). Mouse *lincRNA-Cox2* is homologous to the human locus that contains p50-associated cyclooxygenase 2 extragenic RNA (*PACER*) and Cox2-divergent lncRNAs (105). The expression of *lincRNA-Cox2* is induced during macrophage activation. Additionally, *lincRNA-Cox2* regulates the expression of immune response genes in a cis- and trans-immune-regulatory manner (46). In mouse macrophages, *lincRNA-Cox2* has been reported to regulate the host antimycobacterial response and restrict mycobacterial intracellular survival (75). The mechanism of *lincRNA-Cox2*-mediated antimycobacterial activity in macrophages remains unclear. COX2 is an enzyme involved in the synthesis of prostaglandins that play an important role in host defense against *M. tuberculosis* infection in macrophages (106). Therefore, one possible mechanism is that *lincRNA-Cox2* regulates the expression of the *Cox2* gene via inhibition of the transcriptional regulator NF-κB (48). In the context of *M. bovis* BCG infection, a mycobacterium strain closely related to *M. tuberculosis*, the expression of *lincRNA-Cox2* is induced via a TLR2-dependent pathway in mouse RAW 264.7 cells. Interestingly, the knockdown of *lincRNA-Cox2* increases *M. bovis* BCG-induced apoptosis in mouse macrophages (78).

### The Lnc-EST12

LncRNA-early secreted parget with a molecular weight of 12 kDa (*Lnc-EST12*) is a recently identified host lncRNA that facilitates *M. tuberculosis* survival in mouse macrophages (76). The expression of *lnc-EST12* is partially downregulated by the *M. tuberculosis* protein EST12 via the JAK2-STAT5a signaling pathway in mouse BMDMs and RAW 264.7 cells during mycobacterial infection. However, *lnc-EST12* downregulates the expression of several antimycobacterial proinflammatory cytokines, including *IL-1β*, *IL-6*, and *CCL5/8*. Additionally, *lnc-EST12* attenuates the activation of the EST12-induced NLRP3 inflammasome and GSDMD pyroptosis-IL-1β immune pathway, which play crucial roles in host immunity in response to *M. tuberculosis* infection. These regulatory roles of *lnc-EST12* in *M. tuberculosis*-infected macrophages occur when it interacts with the transcription factor far upstream of element-binding protein 3 (FUBP3). Interestingly, *lnc-EST12* expression is still partially inhibited by the ΔEST12 *M.tb* strain compared with the uninfected strain in mouse BMDMs in cell culture, suggesting that additional mycobacterial factors suppress *lnc-EST12* expression in addition to EST12 in macrophages (76).

### Other lncRNAs

Autophagy and apoptosis are two critical cellular pathways involved in controlling mycobacterial infection in macrophages (107). Several host lncRNAs have been found to regulate these two pathways. These lncRNAs include *lncRNA-MIAT* (79), *PCEED1B-AS1* (77), *lncRNA-MEG3* (80) and *lncRNA-EPS* (81). *lncRNA-MIAT* is also known as a gene associated with retinoid-IFN-induced mortality 19 (GRIM-19) in mice. It has been studied primarily in the context of cardiovascular diseases, particularly its association with myocardial infarction (heart attack) (108). It was recently reported that the expression of the *lncRNA MIAT* is highly induced in human THP-1 cells infected with *M. bovis* BCG. In the same study, it was reported that *lncRNA-MIAT* represses autophagy and apoptosis by downregulating the miR-665/ULK1 axis in THP-1-derived macrophages in response to *M. bovis* BCG infection (79). Maternally expressed gene 3 (*lncRNA-MEG3*) is a well-studied lncRNA that plays an important role in various biological processes. Several studies have demonstrated that *lncRNA-MEG3* acts as a tumor suppressor. It inhibits cell proliferation and promotes apoptosis, ultimately suppressing the progression of various tumors, such as glioma and hepatocellular carcinoma (109, 110). Moreover, *lncRNA-MEG3* is also expressed in the brain and has been implicated in neurodevelopment and neuronal functions (111, 112). In response to *M. bovis* BCG infection, the expression of *lncRNA-MEG3* was downregulated in human macrophages. Knockdown of *lncRNA-MEG3* increases autophagy and *M. bovis* BCG killing in human THP-1-derived macrophages (80). Compared with the lncRNAs described above, PCED1B antisense RNA 1 (*PCED1B-AS1*) is a newly discovered and less studied lncRNA. *PCED1B-AS1* has been found to facilitate the progression of multiple human tumors, including glioma, pancreatic ductal adenocarcinoma and hepatocellular carcinoma (113). Recently, the expression of *PCED1B-AS1* was reported to be attenuated in CD14^+^ monocytes from patients with active tuberculosis compared with those from healthy individuals. Additionally, *PCED1B-AS1* regulates apoptosis and autophagy by binding to miR-155 as a miRNA sponge in human macrophages (77). Similar to *PCED1B-AS1*, the lncRNA erythroid pro-survival (*lncRNA-EPS*) is also a less-studied lncRNA. In mouse RAW 264.7 cells, *lncRNA-EPS* regulates apoptosis and autophagy via the KNK/MAPK-dependent pathway during *M. bovis* BCG infection (81).

#### Salmonella typhimurium

Salmonella typhimurium (*S. typhimurium*) is a bacterial species belonging to the genus *Salmonella*. It is a pathogenic bacterium that can cause a type of foodborne disease known as salmonellosis in humans and animals. Like *M. tuberculosis*, *S. typhimurium* uses macrophages as primary host cells for their intracellular replication and has evolved various survival mechanisms to evade the host defense system within macrophages, such as inhibiting the fusion of *S. typhimurium*-containing phagosomes with lysosomes (114). In addition to macrophages, *S. typhimurium* adheres to epithelial cells lining the gut and can invade epithelial cells and disrupt tight junctions. Therefore, the interaction between *S. typhimurium* and host cells (such as macrophages and epithelial cells) in the intestine determines the consequences of *S. typhimurium* infection. Host lncRNAs also play essential roles in *host–S. typhimurium* interactions.

#### The lncRNA NeST

LncRNA NeST (Nettoie Salmonella pas Theiler’s, also called Tmevpg1) is a lncRNA gene located adjacent to the IFN-γ-encoding gene in both mice (*Ifng*) and humans (*IFNG*). The lncRNA *NeST* has been identified as an important regulator of immune responses, particularly in the context of T-cell activation (82). The lncRNA *NeST* is expressed primarily in T lymphocytes, including CD4^+^ and CD8^+^ T cells, which play a central role in coordinating immune responses (32). The lncRNA *NeST* has gained attention for its role in regulating the production of IFN-γ, an important cytokine in the immune response to microbial infections. The lncRNA *NeST* functions as an enhancer RNA, which elevates the expression of nearby genes by interacting with transcription factors. In the context of *S. typhimurium* infection, the lncRNA *NeST* interacts with the protein WD repeat domain 5 (WDR5), a component of chromatin-modifying complexes, leading to epigenetic alterations that promote IFN-γ expression in CD8^+^ T cells in mice. As a result, the lncRNA *NeST* contributes to host resistance to *S. typhimurium* infection (82).

#### The *lncRNA NEAT1 variant 2*

The lncRNA *NEAT1* variant 2 (*NEAT1_2*) is a splice variant of the lncRNA *NEAT1* gene that we described above in the context of *M. tuberculosis* infection (100). Similarly, the lncRNA *NEAT1_2* is involved in the formation of the subnuclear structure paraspeckles in mammalian cells. *S. typhimurium* infection significantly induces the expression of the lncRNA *NEAT1_2* in HeLa cells via the release of lncRNA degradation by the RNA exosome, aided by the nuclear exosome targeting (NEXT) complex. A decreased level of the *NEAT1_2* transcript in *S. typhimurium*-infected HeLa cells impaired the expression of immune-related genes, including TNF superfamily member 9 (*TNFSF9*), C-C motif chemokine ligand 2 (*CCL2*), and colony stimulating factor 1 (*CSF1*). Additionally, attenuated expression of the lncRNA *NEAT1_2* increases host susceptibility to *S. typhimurium* infection in HeLa cells (83).

#### Listeria monocytogenes

*Listeria monocytogenes (L. monocytogenes)* is a bacterium that can cause a serious foodborne disease called listeriosis, which particularly infects certain populations, such as pregnant women, newborns, elderly individuals, and people with compromised immune systems. *L. monocytogenes* can be transmitted through the consumption of contaminated food, especially ready-to-eat foods that are not properly cooked or stored. *L. monocytogenes* has evolved mechanisms to invade host cells, particularly epithelial cells and phagocytic cells such as macrophages. It uses specialized proteins, such as listeriolysin O (LLO), to facilitate entry into cells and replication within the host cell cytoplasm. Like *M. tuberculosis* and *S. typhimurium, L. monocytogenes* is capable of replicating within host cells, which allows it to avoid immune detection and contributes to its persistence in the host (115). Host lncRNAs are involved in the intricate crosstalk between host cells and *L. monocytogenes*, determining the consequences of infection.

Several host lncRNAs, including *lincRNA-Cox2*, antisense-interleukin-1 a-subunit (*AS-IL1a*), *Sros1* and *lincRNA-EPS, have been studied in the context of L. monocytogenes infection within host cells* (32, 46, 84–86). The expression of *LincRNA-Cox2* and *AS-IL1a* is induced by *L. monocytogenes* infection within host cells both *in vitro* and *in vivo*. Within *L. monocytogenes*-infected mouse BMDMs, *AS-IL1α* acts as a transcriptional enhancer to recruit RNA polymerase II (RNAPII) to the *IL-1α* promoter, driving *IL-1α* expression (46, 84). In contrast to *LincRNA-Cox2* and *AS-IL1a*, the expression of *Sros1* and *lincRNA-EPS* is downregulated during *L. monocytogenes* infection in mouse BMDMs in cell culture (85, 86). The lncRNA *Sros1* can bind to the mRNA of the transcription factor signal transducer and activator of transcription 1 (*Stat1*) and subsequently prevent its interaction with the RNA-binding protein cytoplasmic activation/proliferation-associated protein-1 (CAPRIN1), which stabilizes the *Stat1* mRNA in mouse macrophages. During *L. monocytogenes* infection, the *lncRNA Sros1* is degraded via the miR-1-mediated pathway in mouse BMDMs. It therefore increases the stability of *Stat1* mRNA and subsequently elevates the expression of immune-related genes that are critical for host defense against bacterial infection (85). *LincRNA-EPS* deficiency increases the expression of proinflammatory cytokine genes (including *IL-6*, *TNFα*, *IL-1β* and *Ccl5*) and inducible nitric oxide synthase (*iNOS*)), as well as the production of nitric oxide (NO), in mouse BMDMs after *L. monocytogenes* infection compared with that in wild-type mouse BMMs. These host factors are important antibacterial molecules within host cells against intracellular bacterial pathogens. As expected, *lincRNA-EPS*-deficient mice are more resistant to *L. monocytogenes* infection than wild-type mice (86).

#### Pseudomonas aeruginosa

*Pseudomonas aeruginosa* (*P. aeruginosa)* is an opportunistic bacterial pathogen that can cause serious infections in individuals with compromised immune systems, such as those with cystic fibrosis, burn patients, and chronic wounds. The infection sites include the respiratory tract, urinary tract, blood, skin and soft tissues (116, 117). Host lncRNAs play important roles in host−pathogen interactions during *P. aeruginosa* infection.

#### The lncRNA MEG3 transcript 4

The lncRNA MEG3 transcript 4 (lncRNA *MEG3-4*) is one of 10 isoforms of the lncRNA *MEG3* in mice. Recently, *P. aeruginosa* lung infection was shown to downregulate the expression of the lncRNA *MEG3-4* in the lungs and livers of C57BL/6 mice via the TLR4/NF-κB-mediated pathway (87). Interestingly, the mice receiving engineered MH-S cells that overexpress lncRNA *MEG3-4* are more susceptible to *P. aeruginosa* lung infection than those receiving MH-S cells with only the empty vector. It was further found that lncRNA *MEG3-4* acts as a miRNA sponge for miRNA-138, which binds to the mRNA of *IL-1β* and downregulates the production of the IL-1β protein. In a separate study, Balloy et al. analyzed the lncRNA expression profile in bronchial epithelial cells that were isolated from patients with cystic fibrosis (CF) or healthy donors and then infected with *P. aeruginosa in vitro* in cell culture for 0, 2, 4 or 6 hr. As a result, they identified 108 unique host lncRNAs that were differentially expressed in *P. aeruginosa*-infected CF bronchial epithelial cells compared with infected non-CF cells (88). Among them, the expression of 12 lncRNAs was differentially regulated at least at two time points, and the expression of two lncRNAs, *LINC00862* and *CTD-2619J13*, was differentially regulated at all four time points after *P. aeruginosa* infection. Interestingly, the expression of the lncRNA *MEG3* was downregulated in CF bronchial epithelial cells compared with non-CF cells at 4 and 6 hr post-*P. aeruginosa* infection.

#### Legionella pneumophila

*Legionella pneumophila (L. pneumophila)* is an intracellular bacterial pathogen that causes legionellosis, a severe and potentially life-threatening pneumonia in humans. *L. pneumophila* enters the human body primarily through the inhalation of aerosols. Like *M. tuberculosis* and *S. typhimurium, L. pneumophila* has evolved the ability to infect and replicate within human cells, particularly within macrophages. After entry into macrophages, *L. pneumophila* prevents the fusion of Legionella-containing phagosomes with lysosomes, which contain enzymes that can degrade invading pathogens. This prevents bacteria from being destroyed and allows them to establish a specialized compartment called the Legionella-containing vacuole (LCV) as a replication niche for intracellular *L. pneumophila* (118, 119). Similarly, host lncRNAs play a critical role in the interaction between *L. pneumophila* and host cells.

#### The lncRNA *MaIL1*

The lncRNA *MaIL1* (also known as *MAILR*) is an intergenic lncRNA in humans. The expression of *MaIL1* is upregulated via a TLR4-dependent pathway in human blood-derived macrophages *in vitro* after *L. pneumophila* infection. Knockdown of the lncRNA *MaIL1* significantly increases *L. pneumophila* survival and replication within human MDMs, which correlates with attenuated production of the type I IFNs IFNα and IFNβ in Legionella-infected macrophages in cell culture (51). Interestingly, *MaIL1* deficiency-associated host defense failure in *L. pneumophila* can be rescued by the addition of exogenous type I IFNs. It was further demonstrated that the lncRNA *MaIL1* interacts with optineurin, a ubiquitin-adapter protein that binds and regulates TBK1 kinase activity. Therefore, the lncRNA *MaIL1* indirectly upregulates the protein kinase TBK1 and the downstream transcription factor IRF3, ultimately resulting in increased expression of type I IFNs in human macrophages in response to *L. pneumophila* infection. The same study revealed that the expression of another lncRNA, *Linc01215*, was also induced in human blood-derived macrophages after *L. pneumophila* infection. However, the role of *linc01215* in the macrophage response to *L. pneumophila* remains to be defined (51).

#### Brucella abortus

*Brucella abortus* (*B. abortus)* is a bacterial pathogen that causes brucellosis, a zoonotic infectious disease that primarily affects animals but can also be transmitted to humans. *B. abortus* enters the host body through mucosal surfaces, such as the respiratory or digestive tract. Once inside, the bacterium is taken up by phagocytic cells such as macrophages. Like *M. tuberculosis*, *B. abortus* can evade destruction by inhibiting phagolysosome maturation within macrophages. Additionally, *B. abortus* induces the formation of a specialized membrane-bound compartment called the “Brucellosome”. This compartment is derived from the endoplasmic reticulum (ER) and provides an environment favorable for bacterial replication (120).

#### The lncRNA Gm28309 and P33714

The lncRNA *Gm28309* in mice is an ortholog of the human lncRNA *P33714*. By analyzing differentially expressed lncRNAs in *B. abortus*-infected human THP-1 cells (*vs.* uninfected cells) via the human long noncoding RNA V3.0 gene chip, Deng et al. identified 235 differentially expressed lncRNAs (171 upregulated and 64 downregulated) at 24 hr after *B. abortus* infection (89). Six lncRNAs, namely, *P662*, *P30159*, *P16218*, *P3852*, *P33714* and *P12873*, were predicted to be involved in the inflammation pathway via KEGG pathway analysis. Among them, the expression of *P33714* was significantly downregulated in *B. abortus*-infected human THP-1 cells compared with that in uninfected cells. Additionally, the knockdown of *P33714* increased the expression of the cytokines *IL-1β* and *IL-18* and impaired *B. abortus* intracellular survival in human THP-1 cells. Similarly, the expression of the mouse ortholog of *P33714*, *Gm28309*, was also downregulated in *B. abortus*-infected mouse macrophage line RAW 264.7 compared with that in uninfected cells. Additionally, overexpression of *Gm28309* inhibited the activation of the NLRP3 inflammasome and the expression of *IL-1β* and *IL-18* in *B. abortus*-infected mouse RAW 264.7 cells. This study further proposed that *Gm28309* downregulates NLRP3 inflammasome activation likely via two regulatory mechanisms: i) *Gm28309*-mediated reduction in TGF-β production attenuates the activation of TAK1 and IKK kinases and subsequent p65 phosphorylation; and ii) *Gm28309* acts as a miRNA sponge for miR-3068-5p, which releases kB-Ras2 from NF-κB and promotes p65 phosphorylation. In both cases, the activated NF-κB pathway is required for the activation of the NLRP3 inflammasome (89).

#### The LncRNA IFNG-AS1

Interferon gamma antisense RNA 1 (lncRNA *IFNG-AS1*, also known as TMEVPG1 or NeST), as we described above, is a lncRNA that is transcribed from the opposite strand of the gene encoding IFN-γ. The lncRNA *IFNG-AS1* regulates immune responses in the host during microbial infections, including enhancing the expression of *INF-γ* (121, 122). Compared with those in healthy control cells, the expression of the lncRNAs *IFNG-AS1* and *IFN-γ* in peripheral blood mononuclear cells (PBMCs) isolated from patients with brucellosis is highly upregulated. Furthermore, it is correlated with increased production of IFN-γ in the serum of patients with brucellosis, suggesting an antibacterial activity of the lncRNA *IFNG-AS1* in response to *B. abortus* infection (123).

## LncRNAs as diagnostic biomarkers in bacterial infections

LncRNAs have shown great potential as diagnostic biomarkers for various diseases because of their tissue-specific expression patterns, stability, and dysregulation in different pathological conditions. Moreover, lncRNAs have been detected in various human body fluids, including blood, urine, saliva, cerebrospinal fluid, seminal fluid and breast milk (124–128). Their presence in these fluids makes them attractive candidates for noninvasive diagnostic and prognostic biomarkers for infectious diseases, especially for those that are challenging to diagnose due to atypical symptoms, limited access to health care and/or the lack of appropriate diagnostic tools. For example, active TB patients have nonspecific symptoms such as cough, fever, and weight loss, which can be mistaken for other respiratory diseases. Traditional diagnostic methods include sputum smear microscopy and mycobacterial culture-based tests. However, sputum smear microscopy may have low sensitivity, and culture-based methods take time. Advanced diagnostic techniques, the GeneXpert MTB/RIF test and the interferon-gamma release assay (IGRA) are becoming more accessible but may not be available in all health care settings, especially in high-burden TB developing countries. Additionally, the GeneXpert MTB/RIF test and IGRA have limited accuracy in TB patients coinfected with HIV, children and extrapulmonary TB patients (129). Therefore, novel and cost-effective diagnostic tools are urgently needed to meet the WHO End TB Strategy, which targets a 90% reduction in patients suffering from TB and a 95% reduction in deaths from TB by 2035 (130).

An increasing number of studies have demonstrated that the expression levels of host lncRNAs are differentially regulated in *M. tuberculosis*-residing macrophages and the blood of TB patients, indicating the potential application of host lncRNAs as novel biomarkers for TB diagnosis and treatment outcomes. Chen et al. analyzed the lncRNA expression profile in the plasma of 33 TB patients and 11 healthy control subjects via the Arraystar Human LncRNA Microarray V3.0 and identified 511 differentially expressed lncRNAs (163 upregulated and 348 downregulated) in TB patients, such as *NR_038221*, *NR_003142*, *ENST00000570366* and *ENST00000422183*. Pathway analysis revealed that these differentially expressed lncRNAs were enriched mainly in the regulation of alpha-beta T-cell activation and the T-cell receptor signaling pathway (131). A similar result was observed in serum exosomes isolated from active TB patients and healthy individuals. By using a public GEO dataset (GSE94907), Fang et al. reported that the abundance of 9 lncRNAs was differentially regulated in serum exosomes isolated from active TB patients compared with exosomes from healthy individuals (132). Among them, four lncRNAs, *NONHSAT101518.2*, *NONHSAT067134.2*, *NONHSAT148822.1* and *NONHSAT078957.2*, were verified using plasma samples collected from 69 active TB patients and 69 healthy individuals. The results revealed that the expression of all 4 lncRNAs was significantly lower in the plasma of active TB patients than in that of healthy controls. Taken together, these findings suggest that host lncRNAs represent a potential new strategy for TB diagnosis. However, it is possible that these host lncRNAs are also regulated by other microbial infections and are not specific for mycobacterial infection. Further studies are warranted to explore the specificity of host lncRNAs for TB diagnosis, particularly compared with other microbial infections.

To investigate the potential application of host lncRNAs as biomarkers for pulmonary TB, Hu et al. established a diagnostic TB model in combination with a host lncRNA expression profile and patient electronic health records (EHRs) (98). In this study, the lncRNA expression profiles of PBMCs from 7 clinically diagnosed pulmonary TB patients and 5 healthy donors were analyzed via Affymetrix human transcriptome array 2.0 chips. As a result, they identified a total of 325 lncRNAs that were differentially expressed (287 upregulated and 38 downregulated) between clinically diagnosed pulmonary TB patients and healthy controls. The top five lncRNAs were subsequently selected based on the following criteria: a fold change of 2 between two groups, a P value of < 0.05, a signal intensity of > 25, and unreported lncRNAs in the TB literature. This top 5 list includes three upregulated lncRNAs (*n335265*, *ENST00000518552*, and *TCONS_00013664*) and two downregulated lncRNAs (*n333737* and *ENST00000497872*) in pulmonary TB patients compared with the control group. The expression of these five lncRNAs was further evaluated in samples from 141 clinically diagnosed pulmonary TB patients, 159 nontuberculosis disease controls, and 578 healthy individuals. The expression of *n333737*, *ENST00000497872* and *n335265* was differentially regulated in pulmonary TB patients compared with the other two groups, as shown by the microarray results. The remaining two lncRNAs, *ENST00000518552* and *TCONS_00013664*, were excluded from the further modeling step because of their low expression levels. To improve the accuracy of diagnosis, a logistic regression model was trained on three selected host lncRNAs and six EHRs (age, hemoglobin, weight loss, low-grade fever, calcification detected by computed tomography, and IGRA) from the same cohort. The established diagnostic model was validated via a new set of patient data consisting of 97 clinically diagnosed pulmonary TB patients, 140 nontuberculosis disease controls, 392 microbiologically confirmed pulmonary TB patients and 245 healthy individuals. This new diagnostic model works better than the EHR model in discriminating pulmonary TB, including microbiologically confirmed and smear-negative cases (98).

In addition to their application as novel diagnostic biomarkers, host lncRNAs may also be used as criteria for TB treatment outcomes. The host lncRNA *NEAT1* (both *NEAT1_1* and *NEAT1_2*) is highly expressed in PBMCs from patients with pulmonary TB compared with those from healthy individuals. Consistently, the expression of both *NEAT1_1* and *NEAT1_2* is induced by *M. tuberculosis* infection in THP-1-derived macrophages in cell culture. Interestingly, the expression levels of *NEAT1_1* and *NEAT1_2* in the PBMCs of pulmonary TB patients correlate with TB treatment and decline over time (73). A high level of the lncRNA *LOC152742* was also detected in the plasma of active pulmonary TB patients. However, the abundance of the lncRNA *LOC152742* in pulmonary TB patients decreases over the course of TB treatment (133). Similarly, the expression levels of the lncRNAs *n333737* and *ENST00000497872* are significantly increased in pulmonary TB patients who respond well to TB treatment (98).

## Potential application of host lncRNAs as host-directed therapy

RNA-based therapy is a type of medical treatment that utilizes RNA molecules, such as miRNAs, mRNAs and lncRNAs, as therapeutic agents to modulate gene expression, with the goal of treating certain diseases. Currently, 11 RNA-based therapeutics, either small interfering RNAs (siRNAs) or antisense oligonucleotides (ASOs), have been approved by the FDA or the European Medicines Agency (EMA), which target gene expression in the liver, muscle, or central nervous system in human diseases (134). Host lncRNAs have gained significant attention in recent years because of their diverse roles in regulating the cellular response to bacterial infection, as described above. Additionally, many host lncRNAs have structures similar to mRNAs, including poly-A tails and 5’-caps, and both are synthesized by RNA polymerase II. The recent success of mRNA-based vaccines has encouraged the potential application of host lncRNAs as novel therapeutic approaches for infectious diseases (135). In summary, although the therapeutic potential of lncRNAs is still being explored, an increasing number of studies indicate an important role for lncRNAs in the pathophysiology of diseases caused by bacterial pathogens, suggesting their usefulness in host-directed therapies.

## Conclusion and future directions

The insights gained from this review contribute to a deeper understanding of the mechanisms, challenges, and potential avenues for further research in the field. Our examination of the literature has revealed the complexities surrounding the role of host lncRNAs in host−pathogen interactions during bacterial infections, and our understanding of host lncRNAs in bacterial infections is still at an early stage relative to other research fields of lncRNAs, such as cancers, in mammalian cells. However, emerging discoveries, especially with respect to intracellular bacterial pathogens (such as *M. tuberculosis*, *S. typhimurium* and *L. monocytogenes*), as described in this review, have revealed the importance of host lncRNAs in the complex interactions between bacterial pathogens and their hosts. Research on host lncRNAs will enrich our understanding of immune responses and the survival of bacterial pathogens within host cells. Additionally, this growth holds the potential to drive the advancement of host lncRNAs as novel diagnostic and/or host-directed therapies for combatting bacterial infections. Further efforts are clearly needed to precisely delineate the lncRNA-dependent regulatory mechanisms, including the intracellular signaling pathways that regulate the expression of lncRNAs and/or are regulated by lncRNAs and lncRNA-interacting host molecules (such as genomic sites, miRNAs and proteins) within host cells. Furthermore, additional studies are needed to understand the role of lncRNAs in bacteria−host interactions in animal models such as mice.
